# Longitudinal Effects of Immediate and Delayed Estradiol on Cognitive Performance in a Spatial Maze and Hippocampal Volume in Menopausal Macaques Under an Obesogenic Diet

**DOI:** 10.3389/fneur.2020.00539

**Published:** 2020-06-24

**Authors:** Benjamin Zimmerman, Payel Kundu, Zheng Liu, Henryk F. Urbanski, Christopher D. Kroenke, Steven G. Kohama, Cynthia L. Bethea, Jacob Raber

**Affiliations:** ^1^Advanced Imaging Research Center, Oregon Health and Science University, Portland, OR, United States; ^2^Department of Behavioral Neuroscience, Oregon Health and Science University, Portland, OR, United States; ^3^Beckman Institute for Advanced Science and Technology, University of Illinois at Urbana-Champaign, Urbana, IL, United States; ^4^Division of Neuroscience, Oregon National Primate Research Center, Beaverton, OR, United States; ^5^Division of Reproductive and Developmental Science, Oregon National Primate Research Center, Beaverton, OR, United States; ^6^Department of Obstetrics and Gynecology, Oregon Health and Science University, Portland, OR, United States; ^7^Departments of Neurology and Radiation Medicine, Oregon Health and Science University, Portland, OR, United States

**Keywords:** menopause, hormone replacement therapy, obesogenic diet, neurodegeneration, aging

## Abstract

The consumption of a diet high in fat and refined sugars has several health risks, including the development of cognitive decline and neurodegeneration. For women, menopause carries additional health risks that may interact with a high-fat diet in negative ways. Some symptoms of menopause, including cognitive impairments, can be modulated by hormone replacement therapy (HRT), but the hormonal formulation and the timing of the treatment relative to the onset of menopause are critical factors determining its efficacy. Little is known about how obesogenic, high-fat, high-sugar diets interact with HRT in menopause to affect cognition and neurodegeneration. Given the high prevalence of the consumption of an obesogenic Western-style diet, understanding how the effects of HRT are modulated by an obesogenic diet is critical for developing optimized therapeutic strategies for peri- and post-menopausal women. In this study, we investigated by magnetic resonance imaging (MRI) the effects of either immediate or delayed estradiol hormone therapy on cognition and neuroanatomy following ovo-hysterectomy (OvH) of aged, female rhesus macaques on an obesogenic diet. The macaques were followed for 2.5 years after ovo-hysterectomy, with four time points at which anatomical MRIs were acquired. Analysis of hippocampal volumes revealed an interaction between time point and treatment; hippocampal volumes in the delayed estrogen group, but not the immediate estrogen group, increased over time compared to those in untreated controls. Performance on a hippocampal-dependent spatial maze task showed improved performance in estrogen treated animals compared to OvH macaques given placebo. These results indicate that HRT may contribute to beneficial cognitive outcomes after menopause under an obesogenic diet.

## Introduction

Menopause is associated with a cessation of the production of oocytes, as well as a decline in the production of endogenous estrogens and progesterone (1). This can result in a variety of physiological changes, including adverse changes to mood and cognitive effects such as effects on memory (1, 2). Given current average lifespan, most women will live upwards of one third of their lives in a post-menopausal state (3, 4). Hormone replacement therapy (HRT) was prescribed to alleviate the adverse symptoms of menopause for several decades. However, the publication of the findings from the Women's Health Initiative (WHI) in 2002 led to a decrease in HRT usage (3, 5). The results from WHIMS, the cognitive arm of the WHI, indicated that HRT (estrogens with or without progesterone) actually increased the risk of dementia and led to worse cognitive outcomes than post-menopausal women taking placebo (6). However, since the publication of the WHI results, it has become increasingly clear that the length of the period between the menopausal transition and the start of HRT, the critical period hypothesis, is an important factor in determining the outcome of treatment with longer delays being associated with less beneficial HRT outcomes. Participants were on average 10–12 years into menopause at the beginning of the WHI study (3, 7). Systematic reviews on the effects of HRT have yielded mixed results (8–11). This seems due in large part to the heterogeneity between HRT studies, highlighting the importance of investigating specific modulating factors.

Diet is known to be a critical factor in determining health outcomes. Specifically, a diet high in saturated fatty acids (SFAs) and refined sugars, has been shown to contribute substantially to the obesity epidemic as well as to the development of cognitive impairment and dementia (12, 13). This Western-style diet (WSD) has also been specifically associated with impairments in spatial learning and memory (12). Recent estimates indicate that 71.6% of American adults are overweight or obese, and that most Americans are eating more than the recommended amount of dietary fat and refined sugars (14, 15). It is important to note that most of the animal studies conducted to date investigating the effects of a WSD on cognition involved only male animals, highlighting the need to investigate how a WSD impacts health and cognition in female animals. Little is known about how WSDs interact with hormone replacement therapy in menopause. There is some evidence indicating that the effectiveness of HRT on behavioral and cognitive performance is modulated by an obesogenic diet in macaques (16) as well as in rodent models (17), but the mechanisms that may contribute to this diet-HRT interaction on cognition are not known.

The close phylogenetic relationship between non-human primates (NHPs) and humans is reflected in the similarities in neuroanatomy, cognition, and reproductive features in the two groups. Specifically, for work on reproductive senescence, NHPs provide several advantages over rodents as model animals. In both NHPs and humans, reproductive senescence in driven by the depletion of the follicular pool (18, 19). In contrast, rodent reproductive senescence is driven by dysregulation of the hypothalamic-pituitary-ovarian (HPO) axis, resulting in mature viable follicles that are present throughout the lifespan (4). This is in marked contrast to the complete depletion of ovarian follicles seen in primates after reproductive senescence (4). The shared mechanism of reproductive senescence between NHPs and humans, in addition to the relatively long lifespan of NHPs compared to rodents, makes NHP studies especially valuable for determining the effects of HRT on physiological systems in humans. Reproductive senescence in rhesus macaques has been characterized more extensively than in any other NHP (20). The current study involved aged animals rather than surgically menopausal young animals, thus making the results of this work more relevant to peri- and post-menopausal women.

There are many similarities between functional and anatomical correlates of aging in macaques and humans that make rhesus monkeys a valuable post-menopausal model to study cognitive aging. Cognitively, both species show particular patterns of impairment with age-related changes in executive functions, spatiotemporal memory, and recognition memory during healthy aging in the absence of dementia (21–23). In addition to these overall age-related cognitive declines, additional menopause-related cognitive impairments have been observed in both human and non-human primates after accounting for the effects of age (21, 24).

The gross anatomical patterns of aging share both similarities and differences between species. In the frontal cortex, decreases in the volumes of area 46 (part of the dorsolateral prefrontal cortex) and the anterior cingulate cortex have been observed in rhesus macaques using MRI (22). In the delayed nonmatching-to-sample (DNMS) visual recognition memory task, lower volume in both area 46 and the anterior cingulate cortex predicted worse performance on the DNMS task (23). These patterns overlap with gross volumetric declines in prefrontal areas that accompany aging in humans (25, 26).

In humans, hippocampal volume seems to be particularly susceptible to aging, even in the absence of pathology (27, 28). In contrast, hippocampal volumes have not been shown to decrease with age in rhesus monkeys (22, 29). Interestingly, Shamy et al. demonstrated that hippocampal volume predicted acquisition of a delayed response spatiotemporal task in aged rhesus macaques despite observing no significant age-related changes in hippocampal volume (22). Indeed, there are changes in hippocampal structure which predict cognitive performance. Hara et al. observed that, independent of age, synaptic density in the outer molecular layer of the dentate gyrus was lower in peri/post-menopausal rhesus macaques than premenopausal macaques (30). Further, the synaptic density in this area predicted performance on a recognition memory task (30).

The goal of the current study was to investigate the role of treatment timing as well as diet on the effects of HRT on spatial cognition and pertinent brain volumes. We investigated the time required to acquisition of a spatial maze task in aged, postmenopausal rhesus macaques treated with estrogen compared to untreated macaques kept under an obesogenic diet. Further, we analyzed how the volumes of the hippocampus, prefrontal cortex, amygdala, and motor cortex changed over the course of 2.5 years in these same monkeys, who were either treated with HRT immediately after surgically-induced menopause, with HRT after a 2-year delay, or with a placebo control.

## Methods

### Subjects

The animals and experimental treatment paradigm are as described previously by Coleman et al. (16). Briefly, aged female rhesus macaques of 17 years old or older were socially housed in groups of 2–4 animals throughout the study in indoor pens (~3.7 × 2.1 × 2.1 m) and provided with enrichment such as toys and foraging devices. Animals were maintained under fixed photoperiods comprising 12 h of light and 12 h of darkness per day (12L:12D). The activity patterns of these animals have been previously described (31, 32). Monkeys were maintained on a standard laboratory chow (Lab Diet, Inc., St. Louis, MO) before being switched to a WSD at the start of the study (TAD; Lab Diet). Macronutrient composition of the diets is listed in Table 1. Water was provided *ad libitum*. Monkeys were fed the WSD for ~6 weeks before being ovohysterectomized (OvH). The intended length of the study was 3 years; however, after 2.5 years it was necessary to terminate the study due to the manifestation of age-related pathologies.

**Table 1:** Macronutrient profile of standard chow and the WSD.

	**Fat (%)**	**Carbohydrates (sugars)**	**Protein (%)**
Standard Chow	13	69% (6%)	18
Western-style diet	36	44% (18.5%)	18

### Surgery

Monkeys were OvH via a laparotomy procedure by surgical personnel at the Oregon National Primate Research Center (ONPRC). Animals were removed from the group, sedated with ketamine (10 mg/kg) and transported to the surgical site. Animals were returned to their social group after the procedure.

### Treatment

The macaques received either placebo for 30 months, estrogen immediately upon hysterectomy for 30 months, or placebo for 24 months followed by delayed estrogen treatment for an additional 6 months. Estrogen treatment was administered via the implantation of Silastic capsules subcutaneously into the periscapular region. The implants were intended to achieve serum estrogen concentrations between 70 and 100 pg/ml. Placebo-treated animals were implanted with an empty Silastic capsule. Serum estrogen levels were measured every 2 months to ensure that serum estrogen levels stayed within the desired range.

### Spatial Maze Training

The dependent variable for performance on the spatial maze was trials to criterion. Performance was codified as shown in Table 2. Criterion was defined as reaching a behavioral score of 6. Only animals that achieved a score of 6 or above during the course of training were included in the analysis. Spatial maze training took place before the start of hormone treatment in the delayed estrogen group, thus for data analysis, the placebo and delayed estrogen groups were combined into the control group. Out of 8 animals in the estrogen group, 5 reached criterion. Out of 15 animals in the combined control group, 9 reached criterion. Macaques were allowed to search a series of boxes for treats which consisted of M&M's®, craisins®, dried mango, or marshmallows. Treats were initially placed in various locations on the floor of the testing room, then placed on the floor close to a box, then on top of the box, then on the lip of the entry hole of the box, and finally, hidden inside the box.

**Table 2:** Behavioral scoring criteria for the spatial maze task.

**Score**	**Performance Indicator**
0	Failure to eat or pick up any treats
1	Picking up treats within the room
2	Picking up treats from below the box
3	Reaching to top of box and removing treat
4	Reaching to lip of box and removing treat (large hole)
5	Reaching to lip of box and removing treat (small hole)
6	Reaching into box and removing treat
7	Reaching into box and removing treat more than once consecutively
8	Reaching into box and removing treat more than once consecutively without searching any other boxes prior to reaching treat

### Magnetic Resonance Imaging

#### MRI Data Acquisition

Images were acquired at four time points throughout the study: (1) at baseline (before treatment); (2) after 1 year; (3) after 2 years; and (4) after 2.5 years. For each imaging session, the subject was anesthetized using ketamine (15 g/kg) and transferred to the ONPRC MRI Core Facility. The subject was then intubated and continually anesthetized with 1–1.5% isoflurane for the duration of the imaging (≤ 2 h). Animals were physiologically monitored during scans for well-being.

Images were acquired *in vivo* using a Siemens Tim Trio whole body 3T system (Erlangen, Germany) with a 15-channel quadrature knee coil. For each subject at each time point, four T_1_-weighted magnetization-prepared rapid acquisition gradient echo (MPRAGE) images were acquired with the following parameters: TR = 2500 ms, TE = 3.86 ms, TI = 1100 ms, Flip angle = 12 deg, Voxel size = 0.5 × 0.5 × 0.5 mm^3^, FOV = 128 × 128 mm^2^, Matrix size = 256 × 256.

#### MRI Analysis

Preprocessing of the T_1_-weighted MPRAGE images was implemented for all imaging sessions. At the beginning, four T_1_-weighted images collected for the same subject at the same time point were merged as the final anatomical image after motion correction and intensity bias correction. The motion correction was implemented by the rigid-body alignment with the first acquired MPRAGE image using “antsRegistrationSyN.sh” tool from Advanced Normalization Tools (ANTS) software (33). After motion correction, the intensity bias field of each image was corrected using a B-spline approximation routine and hierarchical optimization scheme with the “N4BiasFieldCorrection” tool also in ANTS (34). Then, all four corrected images were averaged as the final image for structural analysis. Next, the baseline merged T1-weighted images were B-spline nonlinearly registered to a head image from the INIA19 template (35) using “antsRegistrationSyN.sh.” Using the resulting transformation parameters, the INIA19 brain mask was reversely mapped to each subject's original space to generate the brain mask using a nearest neighbor interpolation method. For the following time points, the brain masks were generated using the same method but updated references with the corresponding merged image of the same subject at the previous time point. Based on brain masks, skull-stripping of all merged MPRAGE images was achieved and the process of intensity bias correction for all brain images was repeated using the obtained brain masks to limit the correction region to improve correction quality.

After the preprocessing of all MPRAGE images, all baseline brain images were nonlinearly registered to the INIA19 brain template image. According to the transformation parameters, the label map of the INIA19 template, i.e. NeuroMaps, was inversely mapped to each individual original space using nearest neighbor interpolation method. Subsequently, brain image from each subject's next imaging session was registered to their previous imaging session, and then the inversed mapping of the label maps from the previous imaging session using the resulting registration parameters were implemented to generate the label maps for the next imaging session. All volumetric analysis was based on the acquired label maps for each subject.

**Figure 1:**
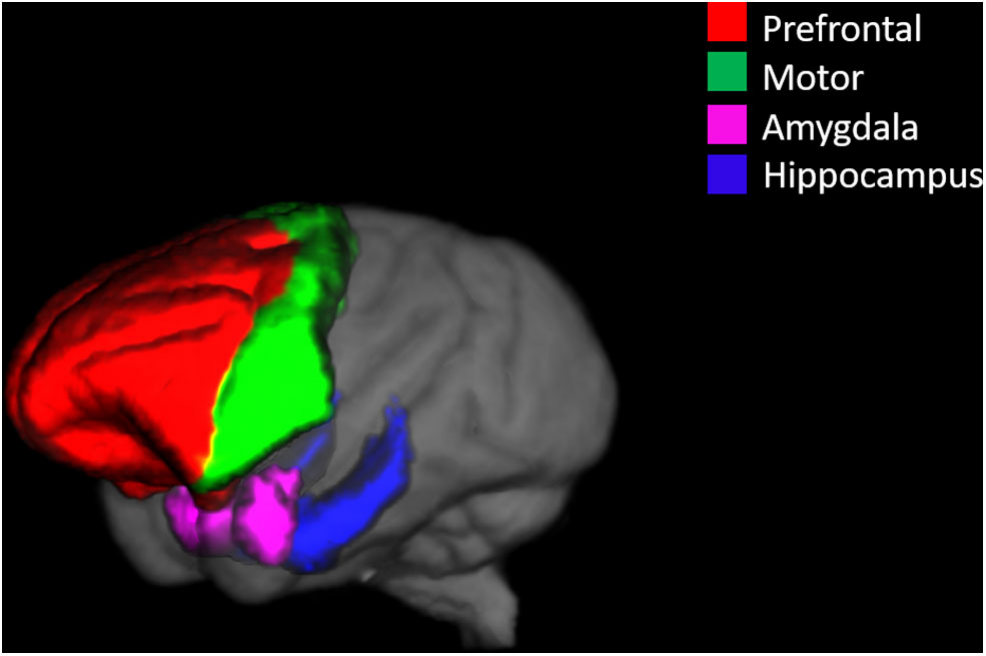
An example glass brain of an individual monkey with the predefined ROIs. The prefrontal ROI is highlighted red, the motor ROI is highlighted green, the amygdala ROI is highlighted magenta, and the hippocampal ROI is highlighted blue.

Because of sample size of this study, we defined four well-resolved regions-of-interest (ROIs) from the smaller parcellations included in the NeuroMaps labels. A list of the included structures for each of the ROIs in presented in Table 3. Figure 1 shows the ROI boundaries in the glass brain of an example subject. All of the ROI boundaries were defined *a priori* before any statistical analysis of the volumes. Hippocampal and prefrontal ROIs were defined specifically because of their hypothesized role in spatial maze performance. The amygdala ROI was defined because a previous study on these monkeys (16) observed treatment differences in anxiety-type behaviors which we hypothesized could both affect performance on the task and could manifest anatomically as differential relative amygdala sizes. A motor ROI was defined as a region that we did not expect to be substantially involved in treatment effects on cognitive performance to serve as an anatomical control for the other anatomical analyses. In order to correct for overall head size, the ROIs were normalized by the total brain volume, and these relative volumes were used for all subsequent analysis.

**Table 3:** NeuroMaps labels included in ROI structures.

**Hippocampus**	**Prefrontal**	**Motor**	**Amygdala**
Perforant path	Anterior cingulate gyrus	Precentral gyrus	Lateral amygdalar nucleus
Oriens layer of the hippocampus	Frontal white matter		Accessory basal nucleus of the amygdala
CA fields	Superior frontal gyrus		Basal nucleus of the amygdala
Granular layer of the dentate gyrus	Middle frontal gyrus		Claustral amygdalar area
Molecular layer of the hippocampus	Inferior frontal gyrus		Periamygdalar area
Pyramidal cell layer of the hippocampus	Fronto-orbital gyrus (macaque)		Amygdalar island
Molecular layer of the dentate gyrus	Lateral orbital gyrus		Paralaminar nucleus of the amygdala
Parasubicular area	Straight gyrus		Amygdalohippocampal area
Uncinate gyrus	Medial orbital gyrus		Hippocampal-amygdaloid transition area
Prosubiculum			Amygdala
Subiculum			Central amygdalar nucleus
Presubiculum			Medial amygdalar nucleus
Stratum pyramidale of the CA1 field			Anterior amygdalar area
Stratum pyramidale of the CA3 field			Cortical amygdalar nucleus
Stratum pyramidale of the CA2 field			Amygdala—not otherwise specified
Hilus of the dentate gyrus			
Hippocampal sulcus			
Entorhinal sulcus			
CA1 field			
CA2 field			
Fascia dentata			

*Both left and right labels were included for all bilateral structures*.

### Statistical Analysis

All statistical analyses were completed using the computing environment *R* (version 3.6.0; R Development Core Team, 2019). Significance for all tests performed was defined as an alpha value of ≤ 0.05. *R* packages used for the analysis and presentation of data included: car (3.0.6), dplyr (0.8.1), effects (4.1.3), ggplot2 (3.1.1), ggpubr (0.2), ggsignif (0.6.0), lme4 (1.1.21), lmerTest (3.1.0), psych (1.8.12), and xlsx (0.6.1) (36–44). The *R* code for the analysis is provided in the Supplementary Materials.

Performance on the spatial maze was assessed using two sample *t*-tests to compare the means of trials to criterion between rhesus monkeys given immediate estrogen and the OvH controls. Because trials to criterion may be expected to be count-distributed, we also tested these group differences using a Poisson model. Since the OvH + Delayed E group had still not received estrogen at the time of training in the study, the subjects belonging to this group were combined with the OvH control group for the comparison of performance on the spatial maze training. Because of the temporal proximity to the spatial maze training, relative volumes calculated from the second imaging session (1-year after OvH) were used to predict trials to criterion. Pearson correlations were used to test the relationships between cognitive task variables and the four selected ROI volumes. An overall treatment difference in the number of training days was tested using a one-way ANOVA.

The longitudinal effects of the treatments on each ROI volume were modeled using a linear mixed effect model:

$$\begin{array}{r} Relative\;Volume\;\sim \;Year\;*\;Treatment+\left(1|Subject\right) \end{array}$$

Where random effect intercepts are estimated for the subject, and fixed effects are estimated for the slopes for the year, the treatment factor, and their interaction. *T*-tests and *p*-values were calculated using the Satterthwaite's degrees of freedom method. Since the OvH + Delayed E group did not receive HRT until after 2 years, an additional one-way ANOVA was conducted to assess if there were any group differences on hippocampal volume for the final imaging session only.

## Results

### Spatial Maze

**Figure 2:**
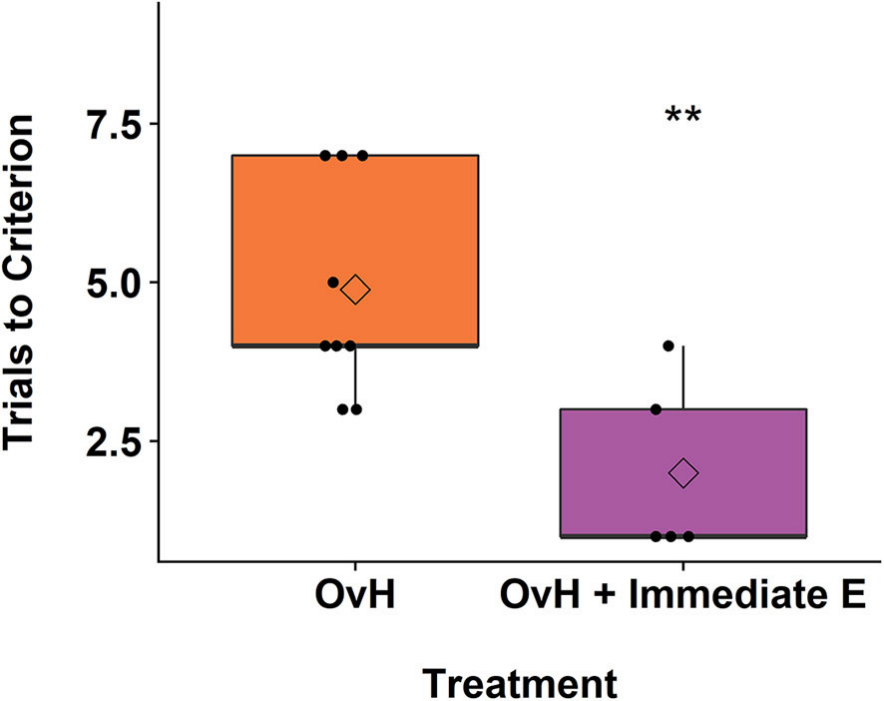
A box plots demonstrating the difference between the OvH and OvH + Immediate E groups in trials to criterion on the spatial maze task. The circles show the scores of individual animals, while the diamonds within the box plots represent the group means. **p < 0.01.

Of the 23 monkeys in the study, only 14 of the monkeys reached criterion on the spatial maze training. Levene's test for homogeneity of variance showed that the variances for trials to criterion were not significantly different between the OvH + immediate E and OvH control groups *F*_(1, 12)_ = 0.195, *p* = 0.667. As illustrated in Figure 2, estrogen treated animals (*M* = *2.00, SD* = 1.41, *n* = 5 animals) compared to OvH animals given placebo only (*M* = 4.89*, SD* = 1.69*, n* = 9), had significantly fewer trials to criterion, *t*_(12)_ = −3.23, *p* = 0.00724. Using a Poisson model, similar results were obtained, *z* = −2.55, *p* = 0.0107.

While there was a significant effect of treatment on the performance during the training of the spatial maze task, there was no relationship between the brain volumes in our chosen ROIs and the spatial maze trials to criterion, (Amygdala: *r*_(12)_ = −0.476, *p* = 0.0851, Hippocampus: *r*_(12)_ = −0.244, *p* = 0.401, Prefrontal: *r*_(12)_ = −0.0284, *p* = 0.923, Motor: *r*_(12)_ = 0.217, *p* = 0.455).

**Figure 3:**
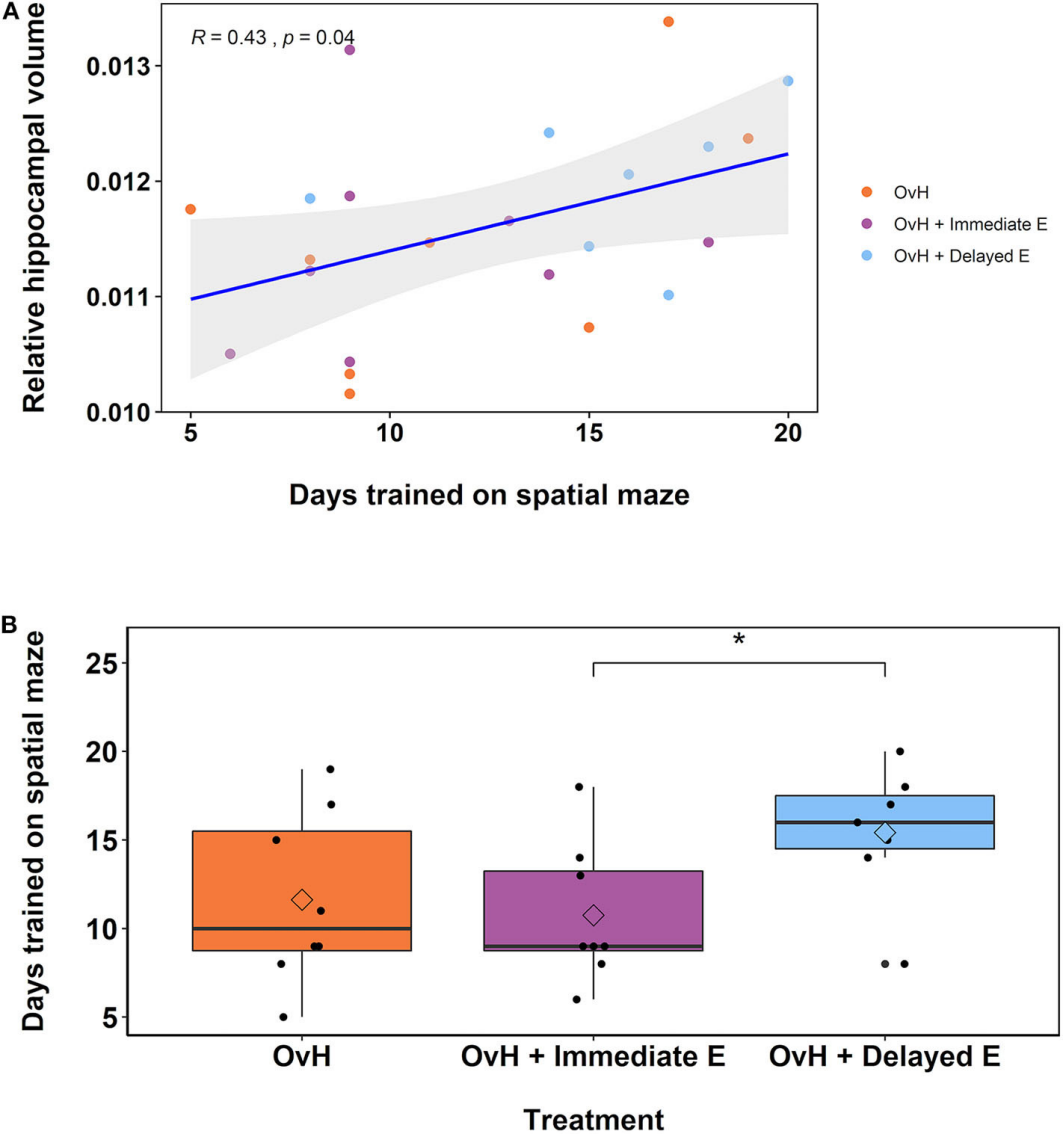
**(A)** There was a significant correlation between the relative hippocampal volumes from the second imaging session and the total number of days trained on the spatial maze task. The animals belonging to the different experimental groups are indicated in distinct colors. **(B)** Box plots demonstrating the difference between the OvH, OvH + Immediate E, and OvH + Delayed E groups with regard to the total number of days trained on the spatial maze task. The circles illustrate the scores of individual animals, while the diamonds within the box plots represent the group means. *p < 0.05 (uncorrected *t*-test). means. *p < 0.05 (uncorrected *t*-test).

Over the course of the spatial maze training, individual monkeys received varying total number of days of training. As shown in Figure 3A, this variation in days trained positively correlated with the relative hippocampal volumes measured in the imaging session following the spatial maze training, *r*_(21)_ = 0.432, *p* = 0.0396. At later imaging sessions, this correlation no longer reached significance, but still showed a trend at 2 years after treatment, *r*_(17)_ = 0.420, *p* = 0.0732, and 2.5 years after treatment, *r*_(14)_ = 0.456, *p* = 0.0756. None of the other chosen ROIs showed a significant correlation with the total number of days of training at the imaging session following training (Amygdala: *r*_(21)_ = 0.190, *p* = 0.386, Prefrontal: *r*_(21)_ = −0.0151, *p* = 0.945, Motor: *r*_(21)_ = −0.383, *p* = 0.0712).

Given the observation that the number of days of training on the spatial maze correlated with the hippocampal volumes, particularly at the 1-year imaging session, we assessed whether there was a difference in the total number of training days by treatment group. As shown in Figure 3B, there was a non-significant, but suggestive effect of treatment group on the total number of days trained, *F*_(2, 20)_ = 2.51, *p* = 0.106. Welch independent sample *t*-tests showed a difference between the OvH + Immediate E and OvH + Delayed E groups, *t*_(12.817)_ = −2.337, *p* = 0.0364, but not between the OvH and the OvH + Delayed E groups, *t*_(12.880)_ = −1.693, *p* = 0.115, or the OvH and the OvH + Immediate E groups, *t*_(13.389)_ = 0.396, *p* = 0.698.

### Treatment Effects on Brain Volumes

**Figure 4:**
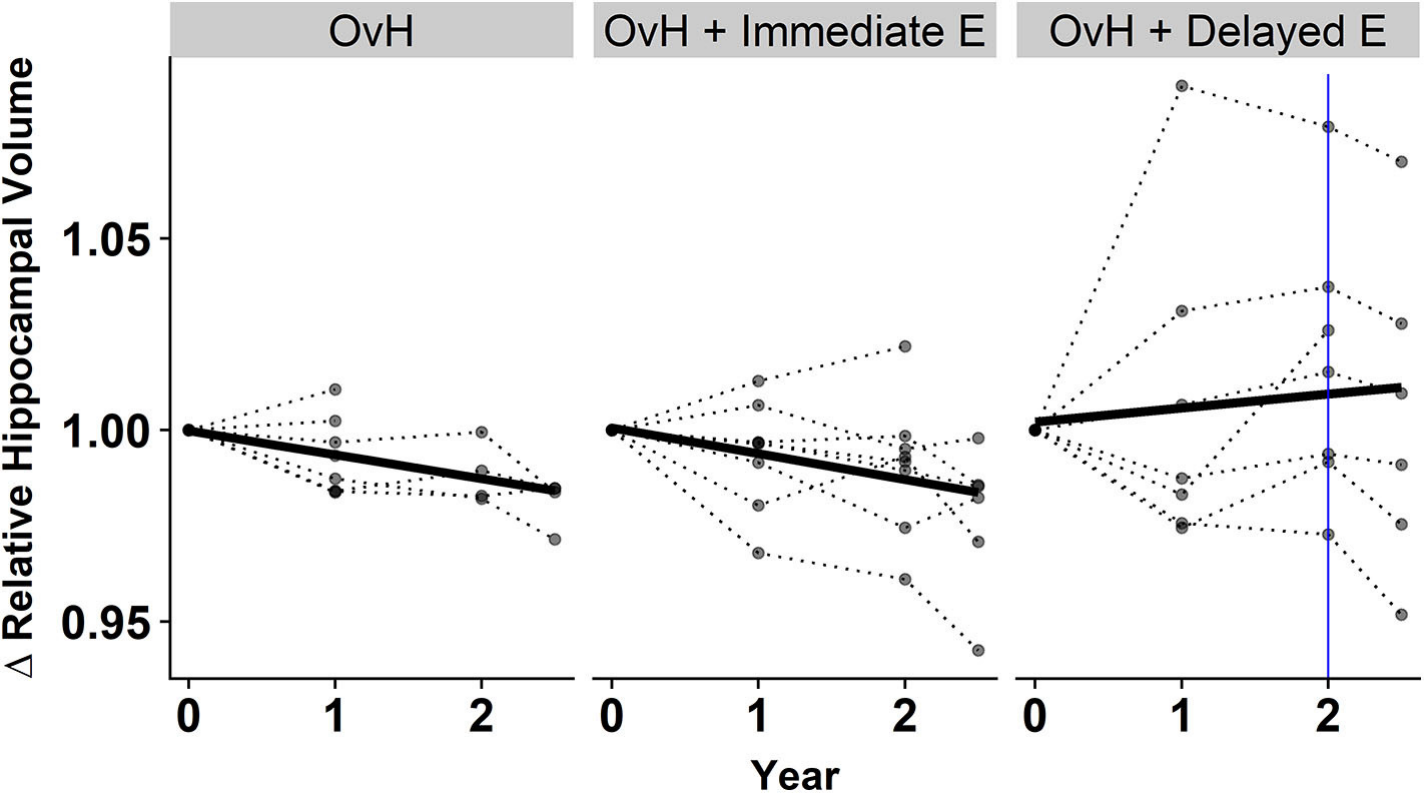
There was a treatment x year interaction for predicting the relative hippocampal volumes from the results of the linear mixed effect model. The relative hippocampal volumes are presented as changes from the baseline hippocampal volumes for each subject. Individual animal trajectories are shown by connecting time points with dotted lines. Each treatment group is showed as its own panel. The blue line in the OvH + Delayed E group shows the time point where HRT began to be administered.

In addition to assessing the relationship between cognitive performance and regional brain volumes, the longitudinal effects of the treatments on brain volume were also modeled. We modeled the time^*^group fixed effects for predicting each of the regional volumes. A significant fixed effect was observed where increased relative hippocampal volume over time was predicted only in the OvH + Delayed E group (*p* = 0.0374, Figure 4, Table 4). An ANOVA on the final imaging session data, after the OvH + Delayed E group received HRT, did not show overall group differences, *F*_(2, 13)_ = 2.106, *p* = 0.161.

**Table 4:** Linear mixed-effect model results predicting relative hippocampus volumes.

**Fixed effects**	**Estimate**	**Standard Error**	**df**	***t* value**	**Pr (>|t|)**
(Intercept)	1.152e-02	3.203e-04	2.058e+01	35.956	<2e-16^***^
Year	−6.899e-05	3.949e-05	5.527e+01	−1.747	0.0862
OvH + Immediate E	−8.818e-06	4.529e-04	2.056e+01	−0.019	0.9847
OvH + Delayed E	4.266e-04	4.688e-04	2.056e+01	0.910	0.3734
Year: OvH + Immediate E	−3.476e-06	5.062e-05	5.515e+01	−0.069	0.9455
Year: OvH + Delayed E	1.097e-04	5.143e-05	5.514e+01	2.133	0.0374^*^

* *p < 0.05*,

** *p < 0.01*,

*** *p < 0.001*.

Additionally, time alone significantly predicted increases in both the relative prefrontal (*p* = 0.00301; Table 5) and relative motor (*p* = 0.00290; Table 6) volumes. No fixed effects in this model predicted the relative amygdala volumes, although there was a trend, whereby in the OvH + Delayed E group the relative amygdala volumes increased over time (Table 7).

**Table 5:** Linear mixed-effect model results predicting relative prefrontal volumes.

**Fixed effects**	**Estimate**	**Standard Error**	**df**	***t* value**	**Pr (>|t|)**
(Intercept)	1.110e-01	1.424e-03	2.143e+01	77.970	<2e-16^***^
Year	7.887e-04	2.542e-04	5.575e+01	3.102	0.00301^**^
OvH + Immediate E	2.846e-03	2.013e-03	2.140e+01	1.414	0.17178
OvH + Delayed E	1.978e-03	2.084e-03	2.139e+01	0.949	0.35317
Year: OvH + Immediate E	−1.222e-04	3.261e-04	5.551e+01	−0.375	0.70933
Year: OvH + Delayed E	−3.954e-04	3.313e-04	5.548e+01	−1.193	0.23782

* *p < 0.05*,

** *p < 0.01*,

*** *p < 0.001*.

**Table 6:** Linear mixed-effect model results predicting relative motor volumes.

**Fixed effects**	**Estimate**	**Standard Error**	**df**	***t* value**	**Pr (>|t|)**
(Intercept)	4.350e-02	9.830e-04	2.067e+01	44.249	<2e-16^***^
Year	3.844e-04	1.233e-04	5.535e+01	3.116	0.0029^**^
OvH + Immediate E	7.622e-04	1.390e-03	2.065e+01	0.548	0.5893
OvH + Delayed E	−3.535e-04	1.439e-03	2.065e+01	−0.246	0.8083
Year: OvH + Immediate E	−1.864e-04	1.581e-04	5.523e+01	−1.179	0.2434
Year: OvH + Delayed E	1.433e-04	1.606e-04	5.522e+01	0.892	0.3764

* *p < 0.05*,

** *p < 0.01*,

*** *p < 0.001*.

**Table 7:** Linear mixed-effect model results predicting relative amygdala volumes.

**Fixed effects**	**Estimate**	**Standard Error**	**df**	***t* value**	**Pr (>|t|)**
(Intercept)	8.971e-03	2.665e-04	2.052e+01	33.665	<2e-16^***^
Year	4.996e-05	3.073e-05	5.525e+01	1.626	0.1096
OvH + Immediate E	−1.623e-04	3.768e-04	2.051e+01	−0.431	0.6712
OvH + Delayed E	−1.586e-04	3.900e-04	2.051e+01	−0.407	0.6884
Year: OvH + Immediate E	−3.933e-05	3.938e-05	5.515e+01	−0.999	0.3223
Year: OvH + Delayed E	7.805e-05	4.001e-05	5.514e+01	1.950	0.0562

* *p < 0.05*,

** *p < 0.01*,

*** *p < 0.001*.

## Discussion

In this study, the effects of estradiol treatment on spatial maze performance in aged OvH macaques on an obesogenic diet were investigated. Additionally, the effects of immediate vs. delayed estrogen treatment on brain anatomy were assessed. Treatment with estradiol improved performance on spatial maze training, as measured by trials to criterion. In addition, average hippocampal volume increased over time in the delayed estrogen group, but not in the immediate estrogen or placebo groups. There was no effect of hormone treatment on volume in any other brain region investigated.

The finding that estrogen treatment improved acquisition efficiency of the spatial task in OvH macaques is supported by a body of previous literature in both rodent and monkey models, indicating that estradiol treatment improves spatial cognition in females deprived of endogenous estrogens (45–50). In aged surgically menopausal rhesus macaques, improvements in aspects of spatial working memory were reported (49, 51). However, while in the rodent literature largely improvements in spatial cognition with estrogen supplementation as well as during the high-estrogen phases of the estrus cycle have been reported [for a review see Korol and Pisani (46)], the monkey literature is more equivocal. In some studies, impaired spatial performance at high estrogen points in the cycle as well as with the maintenance of ovarian hormones in old age is reported (52, 53). This could be due to methodological differences between studies, as well as to the relative small sample sizes typical of nonhuman primate studies where groups consisting of 4–6 individuals are not uncommon.

The current study was performed in aged female macaques, making this model of menopause relevant to human health. Younger macaques have been shown to differ from older macaques in their cognitive changes after surgical menopause, as well as in changes associated with exogenous hormone supplementation after surgical menopause, highlighting the importance of using an age-appropriate model (48, 53).

Recently, Coleman et al. (16) reported on the effects of both delayed and immediate estrogen replacement on behavior performance compared to placebo-controls after OvH in the same aged rhesus macaques under obesogenic diets involved in the present study (16). Their results demonstrated that macaques under an obesogenic diet exhibited increased sedentary and anxiety-like behaviors, but that immediate estrogen replacement promoted activity and ameliorated anxiety-like behavior. In contrast, delayed estrogen (~2 years after surgical menopause) showed equivocal results, highlighting that hormone replacement therapy starting shortly after the cessation of endogenous production of estrogens is more likely to yield health benefits.

The group by session interaction observed in predicting hippocampal volume was somewhat unexpected. The delayed estrogen group showed increasing hippocampal volumes over time compared to the other groups, even though the beneficial effects of HRT would be expected to be stronger with immediate hormone replacement after OvH. A closer look at the individual trajectories in hippocampal volumes (Figure 4) suggests some possible reasons underlying this effect. First, it appears that the volumetric increases between baseline and the year 1 imaging sessions drive the effect of an overall increase over the 2.5 years. However, this increase in volume precedes any HRT in the delayed treatment group. This highlights the discrepancy between the patterns observed between the delayed estrogen group and the OvH control, since neither group had received any estrogen. An analysis of group differences at only the last imaging time point, after the delayed estrogen group had received HRT, showed no overall group differences in relative hippocampal volume, adding further support to the idea that something other than the HRT was driving the difference in hippocampal volumes. This study provides some suggestive evidence that the difference might de due to the amount of training on the task given to the monkeys in the different groups. There was an unexpected suggestive, but non-significant, effect of treatment group on the total number of days the monkeys were trained on the task. There was also a significant relationship between hippocampal volume and the number of days of training on the spatial maze task. Taken together, these results suggest that the difference between the trajectories of hippocampal volumetric change may have more to do with the amount of time spent training on the task than the hormone replacement treatment.

While there was a correlation between the number of days of training and the hippocampal volume, there was no relationship between hippocampal volume and the actual performance on the task. This is an intriguing contrast, suggesting that *changes* in hippocampal volume over time might be more reflective of cognitive training, whereas *current* hippocampal volume might be more predictive of cognitive performance. For example, Shamy and colleagues demonstrated that hippocampal volume predicted acquisition of a delayed response spatiotemporal task, despite finding no evidence of age-related changes in hippocampal volumes (22). Hippocampal volumes have been shown to be particularly plastic to environmental factors over relatively short time periods. For example, in a recent study done on eight expeditioners to a constrained and isolated environment in Antarctica, substantial average decreases in volume of about 7.2% were observed after a 14 month period (54). It is possible that the enrichment from a hippocampally-mediated task could act on hippocampal volumes over the training period and even offset or reverse potential declines from being housed in pens with smaller social units.

Rapid changes in hippocampal volume on the same time scale as those observed here have also been reported in previous animal studies. In mice, over the course of the 4–6 day estrus cycle, changes in hippocampal volume of 2–3%, which are in the range of the changes seen in the present study, have been observed (55). In humans, aerobic exercise increases hippocampal volume transiently after only 6 weeks of the intervention (56). Importantly, both studies found rapid changes in hippocampal volume in adult animals, indicating that adult hippocampal plasticity is sufficient to measurably change the volume of the hippocampus. Additionally, spatial learning can increase the size of the adult hippocampus, as illustrated by Wollett and Maguire's studies in London taxi drivers (57). The changes in hippocampal volume seen in our study could have been driven by the learning aspect of training on the spatial maze, or alternatively by the exercise inherent in the spatial maze training protocol.

Estrogen has known effects on weight due to its actions on food intake suppression (58–60). Additionally, adiposity has been linked to cognitive impairment, especially during aging (61). Thus, body weight could account for some of the cognitive changes seen in estrogen replacement regimens like those described here. These effects are difficult to separate, especially in small samples, and future studies should consider the effects of these modulating factors.

Some limitations of the present study include the generalizability of surgical menopause to natural menopause (however, older animals were used to approximate the equivalent age of clinical menopause), the lack of a non-obesogenic group of animals for comparison, as well as the relative small sample size typical for NHP studies (62). Some previous work has indicated that in humans as well as rodent models, surgical menopause results in different cognitive outcomes, often more adverse, than cognitive outcomes seen in natural or gradual menopause (63, 64). This is likely due to the fact that abrupt loss of hormones seems to be more deleterious for cognition than the more gradual loss seen in natural menopause. However, while natural menopause (as measured by serum estradiol and luteinizing hormone levels, as well as ovarian histology) has been observed in rhesus macaques, it was found in only a subset of studied animals and occurred in their late twenties, which is beyond the average lifespan of this species (65). Thus, surgical menopause in aged macaques is arguably the best alternative. The fact that all of the monkeys were old and under an obesogenic diet precludes the possibility of investigating diet-driven effects on anatomy and make it very difficult to observe any age-related declines in volumes. In addition, larger ROIs were chosen for analysis in order to increase the size of the volumes to look at possible changes and reduce comparisons. However, by choosing just a few ROIs that are relatively large, it is possible that more subtle differences from the smaller, functionally distinct regions that compose the larger regions and/or other brain regions might have been missed.

In summary, this study provides evidence that immediate estrogen replacement after menopause, under an obesogenic diet, contributes to improved performance on the acquisition of a spatial task. Further, this study highlights the potential importance of the course and type of training in predicting gross measures of hippocampal anatomy. This is a particularly critical point, since the training itself and not the performance, was related to hippocampal volume in the present study. Thus, future research on brain health parameters should consider equalizing training time instead of training to a criterion, controlling for the timing of the training, and investigating baseline differences in brain volumes if possible. While the research presented here shows a promising effect of immediate HRT on ameliorating menopause-related cognitive decline under and obesogenic diet, more research is needed to determine the mechanisms driving this effect and its interactions with biological changes resulting from consuming a high-fat, high-sugar diet.

## Data Availability Statement

The datasets generated for this study are available on request to the corresponding author.

## Ethics Statement

The animal study was reviewed and approved by OHSU IACUC committee.

## Author Contributions

JR directed, designed, and oversaw the performed analyses and edited the manuscript. CB and HU obtained funding, designed, and directed the overall study. SK directed and designed the MR imaging of the primates in this study. CK directed and oversaw the preprocessing of the anatomical images. ZL implemented the preprocessing of the images. PK conducted statistical analyses for the spatial maze task and shared the writing of the first draft of the manuscript with BZ. BZ designed and created the ROIs for each monkey, conducted the statistical analyses for the anatomical data, shared the writing of the manuscript with PK, and generated the figures. All authors contributed to the editing of the manuscript, reviewed, and approved the submitted version.

## Conflict of Interest

The authors declare that the research was conducted in the absence of any commercial or financial relationships that could be construed as a potential conflict of interest.
