# Supplementary material for: Longitudinal Effects of Immediate and Delayed Estradiol on Cognitive Performance in a Spatial Maze and Hippocampal Volume in Menopausal Macaques Under an Obesogenic Diet
Source: Front Neurol. 2020 Jun 24;11:539. doi: 10.3389/fneur.2020.00539 (PMC7326936; doi:10.3389/fneur.2020.00539)
Supplement: Supplementary file 1 [file Data_Sheet_1.PDF]

# Longitudinal effects of immediate and delayed estradiol on cognitive performance in a spatial maze and hippocampal volume in menopausal macaques under an obesogenic diet

Ben Zimmerman

March 3, 2020

## Abstract

The consumption of a diet high in fat and refined sugars has several health risks, including the development of cognitive decline and neurodegeneration. For women, menopause carries additional health risks that may interact with a high-fat diet in negative ways. Some symptoms of menopause, including cognitive impairments, can be modulated by hormone replacement therapy (HRT), but the hormonal formulation and the timing of the treatment relative to the onset of menopause are critical factors determining its efficacy. Little is known about how obesogenic, high-fat, high-sugar diets interact with HRT in menopause to affect cognition and neurodegeneration. Given the high prevalence of the consumption of an obesogenic Western-style diet, understanding how the effects of HRT are modulated by an obesogenic diet is critical for developing optimized therapeutic strategies for peri- and post-menopausal women. In this study, we investigated by magnetic resonance imaging (MRI) the effects of either immediate or delayed estradiol hormone therapy on cognition and neuroanatomy following ovo-hysterectomy (OvH) of aged, female rhesus macaques on an obesogenic diet. The macaques were followed for 2.5 years after ovo-hysterectomy, with four time points at which anatomical MRIs were acquired. Analysis of hippocampal volumes revealed an interaction between time point and treatment; hippocampal volumes in the delayed estrogen group, but not the immediate estrogen group, increased over time compared to those in untreated controls. Performance on a hippocampal-dependent spatial maze task showed improved performance in estrogen treated animals compared to OvH macaques given placebo. These results indicate that HRT may contribute to beneficial cognitive outcomes after menopause under an obesogenic diet.

### First load libraries for the analysis of the data

```
library("psych")
library("ggplot2")
```

```
##
## Attaching package: 'ggplot2'
```

```
## The following objects are masked from 'package:psych':
##
##     %+%, alpha
```

```
library("dplyr")
```

```
##
## Attaching package: 'dplyr'
```

```
## The following objects are masked from 'package:stats':
##
##     filter, lag
```

```
## The following objects are masked from 'package:base':
##
##     intersect, setdiff, setequal, union
```

```
library("ggpubr")
```

```
## Loading required package: magrittr
```

```
library("ggsignif")
library("car")
```

```
## Loading required package: carData
```

```
##
## Attaching package: 'car'
```

```
## The following object is masked from 'package:dplyr':
##
##     recode
```

```
## The following object is masked from 'package:psych':
##
##     logit
```

```
library("lme4")
```

```
## Loading required package: Matrix
```

```
## Registered S3 methods overwritten by 'lme4':
##   method                          from
##   cooks.distance.influence.merMod car
##   influence.merMod                car
##   dfbeta.influence.merMod         car
##   dfbetas.influence.merMod        car
```

```
library("lmerTest")
```

```
##
## Attaching package: 'lmerTest'
```

```
## The following object is masked from 'package:lme4':
##
##     lmer
```

```
## The following object is masked from 'package:stats':
##
##     step
```

```
library("effects")
```

```
## lattice theme set by effectsTheme()
## See ?effectsTheme for details.
```

The data is in long form, where each time point is listed as a separate row with up to four time points per subject. A dataset is prepared for each imaging time point to be used for subsequent analysis.

```
setwd("C:/Users/Ben/Desktop")
df <- readRDS(df, file = "frontiers_data.Rda")

first_scan_data <- df[df$Session_.year. == "0",]
second_scan_data <-df[df$Session_.year. == "1",]
third_scan_data <- df[df$Session_.year. == "2",]
last_scan_data <- df[df$Session_.year. == "2.5",]

Group.colors <- c("OvH" = "#F5793A","OvH + Immediate E" = "#A95AA1","OvH + Delayed E" = "#85C0F
9")
```

# Comparison between immediate E and controls in performance on spatial maze training

The first analysis compares the trials to criterion between the OvH + Immediate E group to OvH controls. The OvH + Delayed E group is combined with the OvH group for this first analysis.

```
levels(first_scan_data$Group) <- c("OvH", "OvH + Immediate E", "OvH")
my_comparisons <- list( c("OvH","OvH + Immediate E"))


#tiff("Figure2.tiff", res = 600, units = "in", width = 5, height = 4)


ggplot(first_scan_data, aes(Group, sm_trials_to_criterion, fill=Group))+
  theme_classic() +
  geom_boxplot()+
  geom_jitter(colour = "black",width = .1, height = 0)+
  labs(
       x = "Treatment",
       y = "Trials to Criterion") +

  ylim(0,8) +
  stat_summary(fun.y = mean, geom = "point", shape=23, size =4) +
  theme(legend.position = "none") +
  font("xylab", size = 16, face = "bold") +
  font("xy.text", size = 16, face = "bold", color = "black") +
  theme(panel.border = element_rect(color="black", fill = NA, size = 1)) +
  theme(axis.title.y = element_text(margin = margin(t = 0, r = 20, b = 0, l = 0))) +
  theme(axis.title.x = element_text(margin = margin(t = 20, r = 0, b = 0, l = 0))) +
  stat_compare_means(label = "p.signif", method = "t.test", ref.group = "OvH", size = 7, label.y
= 7.5) +
  stat_summary(fun.y = mean, geom = "point", shape=23, size =4) +
  ylim(NA, 9) +
  scale_fill_manual(values=Group.colors)
```

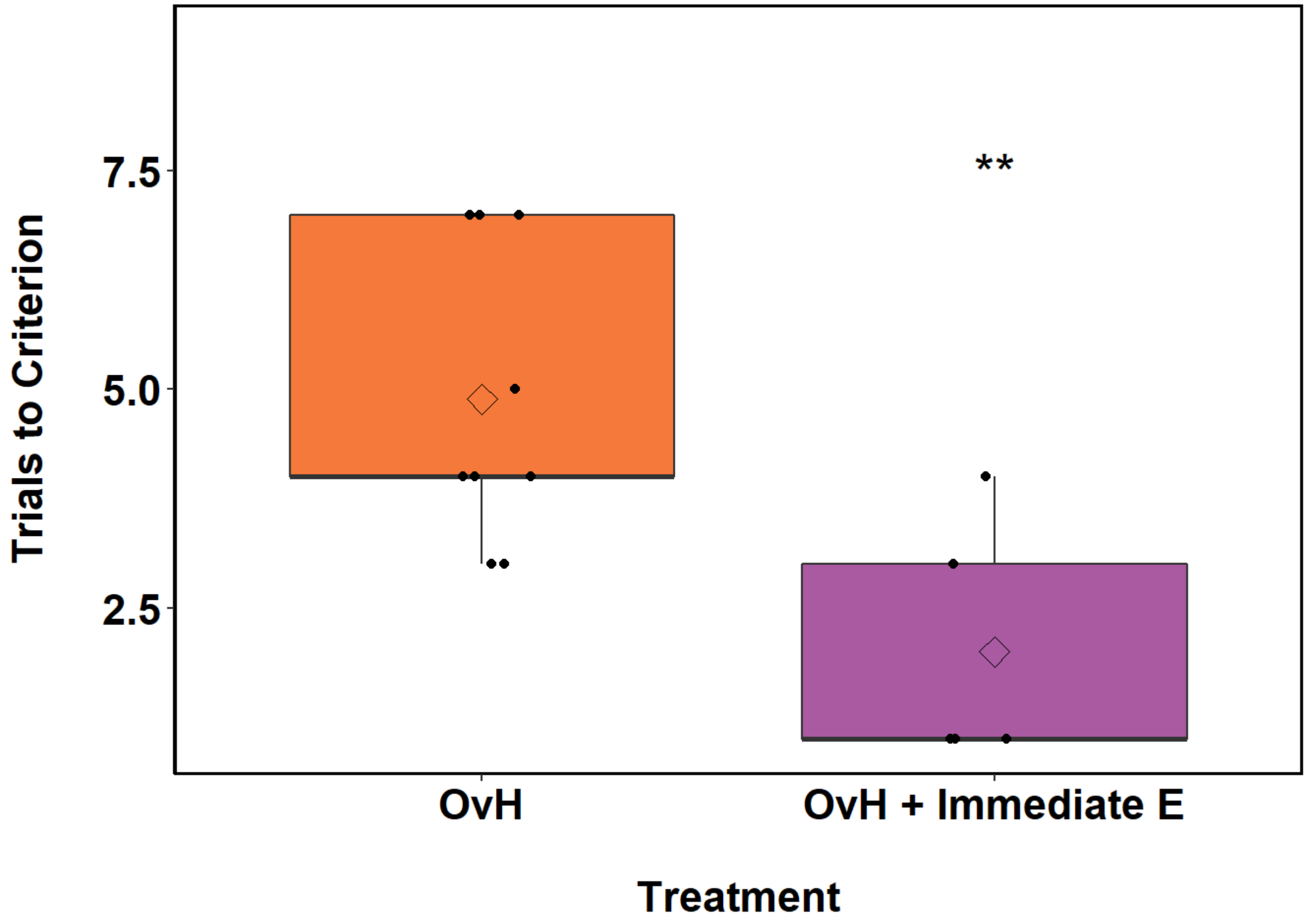


```
#dev.off()
```

```
leveneTest(sm_trials_to_criterion ~ Group, data = first_scan_data)
```

```
## Levene's Test for Homogeneity of Variance (center = median)
##       Df F value Pr(>F)
## group  1  0.1948 0.6668
##       12
```

```
t.test(first_scan_data$sm_trials_to_criterion ~ first_scan_data$Group, var.equal = TRUE)
```

```
## 
##  Two Sample t-test
## 
## data:  first_scan_data$sm_trials_to_criterion by first_scan_data$Group
## t = 3.2282, df = 12, p-value = 0.007243
## alternative hypothesis: true difference in means is not equal to 0
## 95 percent confidence interval:
##  0.9390972 4.8386805
## sample estimates:
##              mean in group OvH mean in group OvH + Immediate E
##                       4.888889                        2.000000
```

# Correlation between number of days trained and hippocampal volume

An analysis was conducted to examine whether the numbers of days trained on the spatial maze task had an effect on the subsequent hippocampal volumes. The figure shows the relationship between the number of days of training and the hippocampal volumes from the second imaging session.

```
scaleFUN <- function(x) sprintf("%.3f", x)

#tiff("Figure3a.tiff", res = 600, units = "in", width = 8, height = 4)

ggscatter(second_scan_data, add = "reg.line", x = "sm_num_days_trained", y = "rel_hipp", size =
2.5, shape = 16, , conf.int = TRUE,  xlab = "Days trained on spatial maze", ylab = "Relative hip
pocampal volume", , color = "Group", palette = c("OvH" = "#F5793A","OvH + Immediate E" = "#A95AA
1","OvH + Delayed E" = "#85C0F9"), add.params = list(color = "blue", fill = "lightgray"), legend
= "right", legend.title = "")   +
  stat_cor(method = "pearson") +
  border() +
  scale_y_continuous(labels = scaleFUN, breaks = c(0.010, 0.011, 0.012, 0.013)) +
  font("xylab", size = 16, face = "bold") +
  font("xy.text", size = 14, face = "bold") +
  theme(axis.title.y = element_text(margin = margin(t = 0, r = 20, b = 0, l = 0))) +
  theme(axis.title.x = element_text(margin = margin(t = 20, r = 0, b = 0, l = 0)))
```

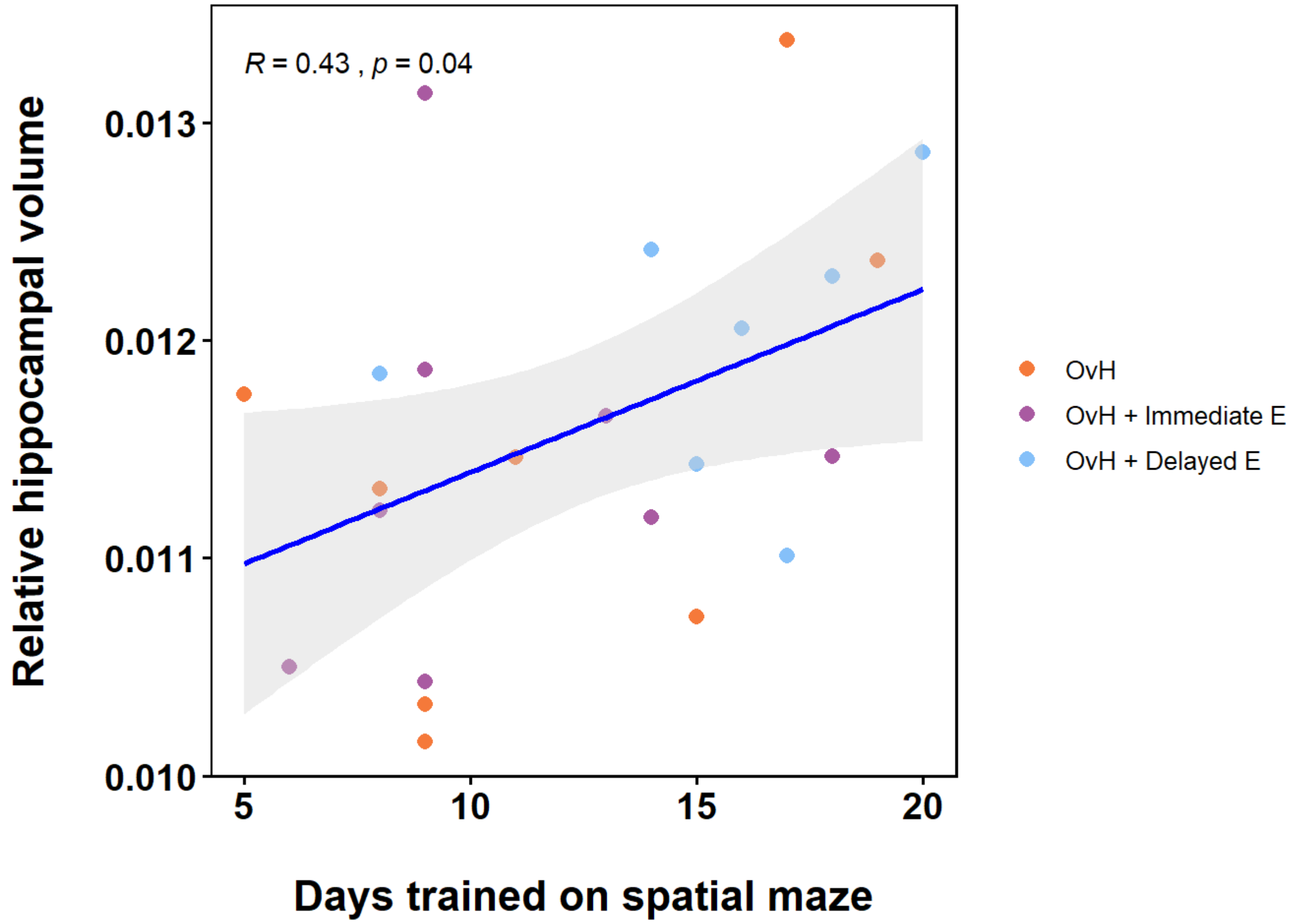


```
#dev.off()
```

We also analyzed the correlation between training and the other predetermined regions-of-interest.

```
  cor.test(second_scan_data$sm_num_days_trained,second_scan_data$rel_hipp, use = "pairwise.compl
ete.obs")
```

```
##
##  Pearson's product-moment correlation
##
## data:  second_scan_data$sm_num_days_trained and second_scan_data$rel_hipp
## t = 2.1947, df = 21, p-value = 0.03957
## alternative hypothesis: true correlation is not equal to 0
## 95 percent confidence interval:
##  0.02400633 0.71655754
## sample estimates:
##       cor
## 0.4319343
```

```
  cor.test(second_scan_data$sm_num_days_trained,second_scan_data$rel_prefrontal, use = "pairwis
e.complete.obs")
```

```
##
##  Pearson's product-moment correlation
##
## data:  second_scan_data$sm_num_days_trained and second_scan_data$rel_prefrontal
## t = -0.069327, df = 21, p-value = 0.9454
## alternative hypothesis: true correlation is not equal to 0
## 95 percent confidence interval:
##  -0.4246809  0.3995669
## sample estimates:
##         cor
## -0.01512669
```

```
  cor.test(second_scan_data$sm_num_days_trained,second_scan_data$rel_amyg, use = "pairwise.compl
ete.obs")
```

```
##
##  Pearson's product-moment correlation
##
## data:  second_scan_data$sm_num_days_trained and second_scan_data$rel_amyg
## t = 0.88531, df = 21, p-value = 0.386
## alternative hypothesis: true correlation is not equal to 0
## 95 percent confidence interval:
##  -0.2413933  0.5582377
## sample estimates:
##       cor
## 0.1896828
```

```
  cor.test(second_scan_data$sm_num_days_trained,second_scan_data$rel_motor, use = "pairwise.comp
lete.obs")
```

```
##
##  Pearson's product-moment correlation
##
## data:  second_scan_data$sm_num_days_trained and second_scan_data$rel_motor
## t = -1.9007, df = 21, p-value = 0.07116
## alternative hypothesis: true correlation is not equal to 0
## 95 percent confidence interval:
##  -0.68685004  0.03454079
## sample estimates:
##        cor
## -0.3831161
```

The relationship between hippocampal volume and the number of days trained reduced, but maintained a trend over the subsequent imaging sessions.

```
  cor.test(second_scan_data$sm_num_days_trained, second_scan_data$rel_hipp, use = "pairwise.comp
lete.obs")
```

```
##
##  Pearson's product-moment correlation
##
## data:  second_scan_data$sm_num_days_trained and second_scan_data$rel_hipp
## t = 2.1947, df = 21, p-value = 0.03957
## alternative hypothesis: true correlation is not equal to 0
## 95 percent confidence interval:
##  0.02400633 0.71655754
## sample estimates:
##       cor
## 0.4319343
```

```
  cor.test(third_scan_data$sm_num_days_trained, third_scan_data$rel_hipp, use = "pairwise.comple
te.obs")
```

```
##
##  Pearson's product-moment correlation
##
## data:  third_scan_data$sm_num_days_trained and third_scan_data$rel_hipp
## t = 1.9094, df = 17, p-value = 0.07324
## alternative hypothesis: true correlation is not equal to 0
## 95 percent confidence interval:
##  -0.04200755  0.73427883
## sample estimates:
##       cor
## 0.4202196
```

```
  cor.test(last_scan_data$sm_num_days_trained, last_scan_data$rel_hipp, use = "pairwise.complet
e.obs")
```

```
##
##  Pearson's product-moment correlation
##
## data:  last_scan_data$sm_num_days_trained and last_scan_data$rel_hipp
## t = 1.9189, df = 14, p-value = 0.07562
## alternative hypothesis: true correlation is not equal to 0
## 95 percent confidence interval:
##  -0.05088527  0.77640813
## sample estimates:
##       cor
## 0.4563306
```

Although the amount of training predicted the hippocampal volume. The hippocampal volume did not predict spatial maze performance in any of our regions-of-interest.

```
  cor.test(second_scan_data$sm_trials_to_criterion,second_scan_data$rel_hipp, use = "pairwise.co
mplete.obs")
```

```
##
##  Pearson's product-moment correlation
##
## data:  second_scan_data$sm_trials_to_criterion and second_scan_data$rel_hipp
## t = -0.87131, df = 12, p-value = 0.4007
## alternative hypothesis: true correlation is not equal to 0
## 95 percent confidence interval:
##  -0.6857553  0.3292656
## sample estimates:
##        cor
## -0.2439286
```

```
  cor.test(second_scan_data$sm_trials_to_criterion,second_scan_data$rel_prefrontal, use = "pairw
ise.complete.obs")
```

```
##
##  Pearson's product-moment correlation
##
## data:  second_scan_data$sm_trials_to_criterion and second_scan_data$rel_prefrontal
## t = 0.098556, df = 12, p-value = 0.9231
## alternative hypothesis: true correlation is not equal to 0
## 95 percent confidence interval:
##  -0.5098334  0.5507088
## sample estimates:
##        cor
## 0.02843903
```

```
  cor.test(second_scan_data$sm_trials_to_criterion,second_scan_data$rel_amyg, use = "pairwise.co
mplete.obs")
```

```
##
##  Pearson's product-moment correlation
##
## data:  second_scan_data$sm_trials_to_criterion and second_scan_data$rel_amyg
## t = -1.8763, df = 12, p-value = 0.08513
## alternative hypothesis: true correlation is not equal to 0
## 95 percent confidence interval:
##  -0.80374487  0.07267202
## sample estimates:
##        cor
## -0.4762717
```

```
  cor.test(second_scan_data$sm_trials_to_criterion,second_scan_data$rel_motor, use = "pairwise.c
omplete.obs")
```

```
##
##  Pearson's product-moment correlation
##
## data:  second_scan_data$sm_trials_to_criterion and second_scan_data$rel_motor
## t = 0.77156, df = 12, p-value = 0.4553
## alternative hypothesis: true correlation is not equal to 0
## 95 percent confidence interval:
##  -0.3540111  0.6706264
## sample estimates:
##       cor
## 0.2174035
```

# Differences in number of days trained between treatment groups

Given the relationship between hippocampal volume and training, we wanted to determine if there were any differences in the training received by the different treatment groups that might bias the longitudinal analysis of group differences. We used an ANOVA to evaluate if there were overall group differences. We also examined the pairwise group comparisons using Welch t-tests.

```
first_scan_data <- df[df$Session_.year. == "0",]
levels(first_scan_data$Group) <- c("OvH", "OvH + Immediate E", "OvH + Delayed E")
my_comparisons <- list( c("OvH + Immediate E","OvH + Delayed E"))
Group.colors <- c("OvH" = "#F5793A","OvH + Immediate E" = "#A95AA1","OvH + Delayed E" = "#85C0F
9")

#tiff("d3_Figure3b.tiff", res = 600, units = "in", width = 8, height = 4)

ggplot(first_scan_data, aes(Group, sm_num_days_trained, fill=Group)) +
  geom_boxplot() +
  geom_jitter(colour = "black",width = .1, height = 0) +
  labs(
      x = "Treatment",
      y = "Days trained on spatial maze") +
  stat_summary(fun.y = mean, geom = "point", shape=23, size =4) +
  theme_classic() +
  theme(legend.position = "none") +
  font("xylab", size = 16, face = "bold") +
  font("xy.text", size = 16, face = "bold", color = "black") +
  theme(panel.border = element_rect(color="black", fill = NA, size = 1)) +
  theme(axis.title.y = element_text(margin = margin(t = 0, r = 20, b = 0, l = 0))) +
  theme(axis.title.x = element_text(margin = margin(t = 20, r = 0, b = 0, l = 0))) +
  geom_signif(comparisons = my_comparisons, map_signif_level = TRUE, y_position = c(25), textsiz
e=7, tip_length = 0.05, test=t.test ) +
  ylim(NA, 26) +
  #Specify colors
  scale_fill_manual(values=Group.colors)
```

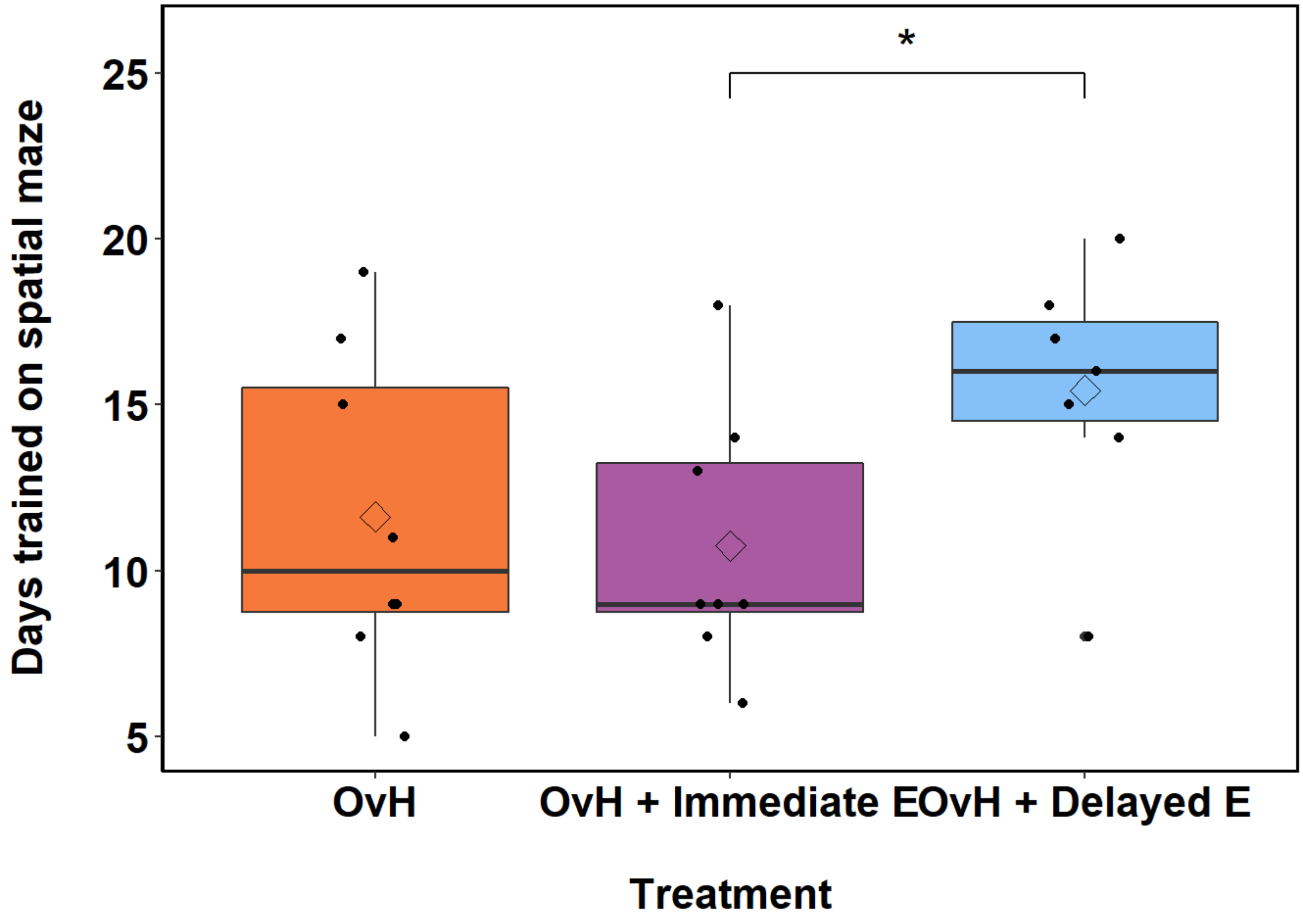


```
#dev.off()
```

```
# One- way ANOVA
Number_days_trained_fit <- aov(sm_num_days_trained ~ Group, data = first_scan_data)
summary(Number_days_trained_fit)
```

```
##             Df Sum Sq Mean Sq F value Pr(>F)
## Group        2   90.6   45.32    2.51  0.106
## Residuals   20  361.1   18.05
```

```
# OvH + Immediate E  vs. OvH + Delayed E
ttest_data_in_plot_one <- first_scan_data %>%
  filter(Group == "OvH + Immediate E" | Group == "OvH + Delayed E") %>%
  select(Group, sm_num_days_trained)

t.test(sm_num_days_trained ~ Group, ttest_data_in_plot_one)
```

```
##
##  Welch Two Sample t-test
##
## data:  sm_num_days_trained by Group
## t = -2.3369, df = 12.817, p-value = 0.03635
## alternative hypothesis: true difference in means is not equal to 0
## 95 percent confidence interval:
##  -9.0099448 -0.3471981
## sample estimates:
## mean in group OvH + Immediate E   mean in group OvH + Delayed E
##                        10.75000                        15.42857
```

```
# OvH vs. OvH + Delayed E
ttest_data_in_plot_two <- first_scan_data %>%
  filter(Group == "OvH" | Group == "OvH + Delayed E") %>%
  select(Group, sm_num_days_trained)

t.test(sm_num_days_trained ~ Group, ttest_data_in_plot_two)
```

```
##
##  Welch Two Sample t-test
##
## data:  sm_num_days_trained by Group
## t = -1.6925, df = 12.88, p-value = 0.1146
## alternative hypothesis: true difference in means is not equal to 0
## 95 percent confidence interval:
##  -8.663216  1.056073
## sample estimates:
##             mean in group OvH mean in group OvH + Delayed E
##                      11.62500                      15.42857
```

```
# OvH vs. OvH + Immediate E
ttest_data_in_plot_three <- first_scan_data %>%
  filter(Group == "OvH" | Group == "OvH + Immediate E") %>%
  select(Group, sm_num_days_trained)

t.test(sm_num_days_trained ~ Group, ttest_data_in_plot_three)
```

```
##
##  Welch Two Sample t-test
##
## data:  sm_num_days_trained by Group
## t = 0.39603, df = 13.389, p-value = 0.6983
## alternative hypothesis: true difference in means is not equal to 0
## 95 percent confidence interval:
##  -3.884164  5.634164
## sample estimates:
##               mean in group OvH mean in group OvH + Immediate E
##                          11.625                          10.750
```

# Analyzing group differences in anatomy

For the anatomical measures, we have four time points following the animals from before their treatment to 2.5 years after the OVX. Since we are missing some data and have longitudinal measures, we used a mixed effect model to control for the within subject random effect. We model the time*group interaction as a fixed effect of interest. Each region-of-interest is considered as a separate model.

```
lmeHIPP <- lmer(rel_hipp ~  1 + Session_.year.*Group + (1 | iD), data = df)
lmeAMYG <- lmer(rel_amyg ~ 1 + Session_.year.*Group + (1 | iD), data = df)
lmeMOTO <- lmer(rel_motor ~ 1 + Session_.year.*Group + (1 | iD), data = df)
lmePREF <- lmer(rel_prefrontal ~ 1 + Session_.year.*Group + (1 | iD), data = df)

summary(lmeHIPP)
```

```
## Linear mixed model fit by REML. t-tests use Satterthwaite's method [
## lmerModLmerTest]
## Formula: rel_hipp ~ 1 + Session_.year. * Group + (1 | iD)
##    Data: df
##
## REML criterion at convergence: -984.3
##
## Scaled residuals:
##     Min      1Q  Median      3Q     Max
## -3.8240 -0.3197  0.0186  0.3035  2.1891
##
## Random effects:
##  Groups   Name        Variance Std.Dev.
##  iD       (Intercept) 8.00e-07 0.0008944
##  Residual             2.62e-08 0.0001619
## Number of obs: 81, groups:  iD, 23
##
## Fixed effects:
##                                         Estimate Std. Error         df
## (Intercept)                            1.152e-02  3.203e-04  2.058e+01
## Session_.year.                        -6.899e-05  3.949e-05  5.527e+01
## GroupOvH + Immediate E                -8.818e-06  4.529e-04  2.056e+01
## GroupOvH + Delayed E                   4.266e-04  4.688e-04  2.056e+01
## Session_.year.:GroupOvH + Immediate E -3.476e-06  5.062e-05  5.515e+01
## Session_.year.:GroupOvH + Delayed E    1.097e-04  5.143e-05  5.514e+01
##                                       t value Pr(>|t|)
## (Intercept)                            35.956   <2e-16 ***
## Session_.year.                         -1.747   0.0862 .
## GroupOvH + Immediate E                 -0.019   0.9847
## GroupOvH + Delayed E                    0.910   0.3734
## Session_.year.:GroupOvH + Immediate E  -0.069   0.9455
## Session_.year.:GroupOvH + Delayed E     2.133   0.0374 *
## ---
## Signif. codes:  0 '***' 0.001 '**' 0.01 '*' 0.05 '.' 0.1 ' ' 1
##
## Correlation of Fixed Effects:
##             (Intr) Sss_.. GOH+IE GOH+DE S_.+IE
## Sessin_.yr. -0.116
## GrpOvH+ImmE -0.707  0.082
## GrpOvH+DlyE -0.683  0.079  0.483
## S_..:GOH+IE  0.090 -0.780 -0.120 -0.062
## S_..:GOH+DE  0.089 -0.768 -0.063 -0.120  0.599
```

```
summary(lmeAMYG)
```

```
## Linear mixed model fit by REML. t-tests use Satterthwaite's method [
## lmerModLmerTest]
## Formula: rel_amyg ~ 1 + Session_.year. * Group + (1 | iD)
##    Data: df
##
## REML criterion at convergence: -1019.2
##
## Scaled residuals:
##     Min      1Q  Median      3Q     Max
## -3.1767 -0.4058 -0.0613  0.4064  1.7831
##
## Random effects:
##  Groups   Name        Variance  Std.Dev.
##  iD       (Intercept) 5.555e-07 0.0007453
##  Residual             1.585e-08 0.0001259
## Number of obs: 81, groups:  iD, 23
##
## Fixed effects:
##                                         Estimate Std. Error         df
## (Intercept)                            8.971e-03  2.665e-04  2.052e+01
## Session_.year.                         4.996e-05  3.073e-05  5.525e+01
## GroupOvH + Immediate E                -1.623e-04  3.768e-04  2.051e+01
## GroupOvH + Delayed E                  -1.586e-04  3.900e-04  2.051e+01
## Session_.year.:GroupOvH + Immediate E -3.933e-05  3.938e-05  5.515e+01
## Session_.year.:GroupOvH + Delayed E    7.805e-05  4.001e-05  5.514e+01
##                                       t value Pr(>|t|)
## (Intercept)                            33.665   <2e-16 ***
## Session_.year.                          1.626   0.1096
## GroupOvH + Immediate E                 -0.431   0.6712
## GroupOvH + Delayed E                   -0.407   0.6884
## Session_.year.:GroupOvH + Immediate E  -0.999   0.3223
## Session_.year.:GroupOvH + Delayed E     1.950   0.0562 .
## ---
## Signif. codes:  0 '***' 0.001 '**' 0.01 '*' 0.05 '.' 0.1 ' ' 1
##
## Correlation of Fixed Effects:
##             (Intr) Sss_.. GOH+IE GOH+DE S_.+IE
## Sessin_.yr. -0.108
## GrpOvH+ImmE -0.707  0.077
## GrpOvH+DlyE -0.683  0.074  0.483
## S_..:GOH+IE  0.084 -0.780 -0.112 -0.058
## S_..:GOH+DE  0.083 -0.768 -0.059 -0.112  0.599
```

```
summary(lmeMOTO)
```

```
## Linear mixed model fit by REML. t-tests use Satterthwaite's method [
## lmerModLmerTest]
## Formula: rel_motor ~ 1 + Session_.year. * Group + (1 | iD)
##    Data: df
##
## REML criterion at convergence: -814.1
##
## Scaled residuals:
##     Min      1Q  Median      3Q     Max
## -3.6734 -0.4906 -0.0427  0.4494  2.9216
##
## Random effects:
##  Groups   Name        Variance  Std.Dev.
##  iD       (Intercept) 7.528e-06 0.0027437
##  Residual             2.556e-07 0.0005056
## Number of obs: 81, groups:  iD, 23
##
## Fixed effects:
##                                         Estimate Std. Error         df
## (Intercept)                            0.0434988  0.0009830 20.6688142
## Session_.year.                         0.0003844  0.0001233 55.3488282
## GroupOvH + Immediate E                 0.0007622  0.0013900 20.6545783
## GroupOvH + Delayed E                  -0.0003535  0.0014387 20.6500541
## Session_.year.:GroupOvH + Immediate E -0.0001864  0.0001581 55.2292352
## Session_.year.:GroupOvH + Delayed E    0.0001433  0.0001606 55.2187117
##                                       t value Pr(>|t|)
## (Intercept)                            44.249   <2e-16 ***
## Session_.year.                          3.116   0.0029 **
## GroupOvH + Immediate E                  0.548   0.5893
## GroupOvH + Delayed E                   -0.246   0.8083
## Session_.year.:GroupOvH + Immediate E  -1.179   0.2434
## Session_.year.:GroupOvH + Delayed E     0.892   0.3764
## ---
## Signif. codes:  0 '***' 0.001 '**' 0.01 '*' 0.05 '.' 0.1 ' ' 1
##
## Correlation of Fixed Effects:
##             (Intr) Sss_.. GOH+IE GOH+DE S_.+IE
## Sessin_.yr. -0.118
## GrpOvH+ImmE -0.707  0.083
## GrpOvH+DlyE -0.683  0.081  0.483
## S_..:GOH+IE  0.092 -0.780 -0.122 -0.063
## S_..:GOH+DE  0.090 -0.768 -0.064 -0.122  0.599
```

```
summary(lmePREF)
```

```
## Linear mixed model fit by REML. t-tests use Satterthwaite's method [
## lmerModLmerTest]
## Formula: rel_prefrontal ~ 1 + Session_.year. * Group + (1 | iD)
##    Data: df
##
## REML criterion at convergence: -719.9
##
## Scaled residuals:
##     Min      1Q  Median      3Q     Max
## -3.1694 -0.4123  0.0200  0.4652  2.3036
##
## Random effects:
##  Groups   Name        Variance  Std.Dev.
##  iD       (Intercept) 1.536e-05 0.003919
##  Residual             1.090e-06 0.001044
## Number of obs: 81, groups:  iD, 23
##
## Fixed effects:
##                                         Estimate Std. Error         df
## (Intercept)                            0.1110254  0.0014240 21.4276488
## Session_.year.                         0.0007887  0.0002542 55.7509193
## GroupOvH + Immediate E                 0.0028463  0.0020131 21.3983309
## GroupOvH + Delayed E                   0.0019775  0.0020835 21.3889415
## Session_.year.:GroupOvH + Immediate E -0.0001222  0.0003261 55.5058137
## Session_.year.:GroupOvH + Delayed E   -0.0003954  0.0003313 55.4841582
##                                       t value Pr(>|t|)
## (Intercept)                            77.970  < 2e-16 ***
## Session_.year.                          3.102  0.00301 **
## GroupOvH + Immediate E                  1.414  0.17178
## GroupOvH + Delayed E                    0.949  0.35317
## Session_.year.:GroupOvH + Immediate E  -0.375  0.70933
## Session_.year.:GroupOvH + Delayed E    -1.193  0.23782
## ---
## Signif. codes:  0 '***' 0.001 '**' 0.01 '*' 0.05 '.' 0.1 ' ' 1
##
## Correlation of Fixed Effects:
##             (Intr) Sss_.. GOH+IE GOH+DE S_.+IE
## Sessin_.yr. -0.168
## GrpOvH+ImmE -0.707  0.119
## GrpOvH+DlyE -0.683  0.115  0.483
## S_..:GOH+IE  0.131 -0.780 -0.174 -0.090
## S_..:GOH+DE  0.129 -0.767 -0.091 -0.175  0.598
```

```
# The change scores were used for presentation in the figure
lmeHIPP_delta <- lmer(rel_hipp_delta ~  1 + Session_.year.*Group + (1 | iD), data = df)
lmeHIPPna <- update(lmeHIPP_delta,na.action=na.exclude)
temp_data <- df
predict(lmeHIPPna)
```

```
##         1         2         3         4         5         6         7 
## 0.9839091 0.9875270 0.9911450 0.9929540 1.0038766 0.9971286 0.9903806 
##         8         9        10        11        12        13        14 
## 0.9870067 0.9997648 0.9935224        NA        NA 0.9963010 0.9900586 
##        15        16        17        18        19        20        21 
##        NA        NA 1.0023758 0.9956279 0.9888799        NA 0.9999834 
##        22        23        24        25        26        27        28 
## 1.0036014 1.0072194        NA 0.9983946 0.9921522 0.9859099 0.9827887 
##        29        30        31        32        33        34        35 
## 0.9947319 0.9884896 0.9822472 0.9791260 0.9903232 0.9939412 0.9975592 
##        36        37        38        39        40        41        42 
## 0.9993682 1.0030603 0.9968179        NA        NA 0.9808743 0.9741264 
##        43        44        45        46        47        48        49 
## 0.9673784 0.9640044 1.0060295 0.9997871        NA        NA 1.0163627 
##        50        51        52        53        54        55        56 
## 1.0199807 1.0235987 1.0254077 1.0027534 1.0063714 1.0099894 1.0117984 
##        57        58        59        60        61        62        63 
## 1.0029782 0.9967358 0.9904935 0.9873723 0.9970266 0.9902787 0.9835307 
##        64        65        66        67        68        69        70 
## 0.9801567 1.0077496 1.0010016 0.9942536 0.9908797 0.9961503 0.9894023 
##        71        72        73        74        75        76        77 
## 0.9826544 0.9792804 0.9752262 0.9788441 0.9824621 0.9842711 1.0463423 
##        78        79        80        81        82        83        84 
## 1.0499603 1.0535783 1.0553873 1.0146898 1.0079419 1.0011939        NA 
##        85        86        87        88        89        90        91 
## 0.9970150 0.9907726 0.9845303 0.9814091 1.0018914 0.9951434 0.9883955 
##        92 
## 0.9850215
```

```
temp_data$fit <- NA
temp_data$fit <- predict(lmeHIPPna)

my.effhipp <- Effect(c("Group","Session_.year."),lmeHIPP_delta)
effhipp <- as.data.frame(my.effhipp)
effhipp$rel_hipp_delta <- effhipp$fit

scaleFUN1 <- function(x) sprintf("%d", x)

#tiff("d2_Figure3.tiff", res = 600, units = "in", width = 7, height = 4)
  ggplot(temp_data, aes(Session_.year., rel_hipp_delta, group= Group)) +
  geom_point(alpha = .5) +
  facet_grid(~Group) +
  theme_bw() +
  geom_line(linetype = "dotted", aes(group=iD)) +
  geom_line(data=effhipp, linetype = "solid", size=1.5 ) +
  labs(
       x = "Year",
       y = expression(bold(paste(Delta," Relative Hippocampal Volume")))) +
  font("xlab", size=14, face="bold") +
  font("xy.text", size=16, face="bold", color = "black") +
  theme(strip.text.x = element_text(size = 15, aes(col=Group)))+
  theme(strip.text.x = element_text(margin = margin(.1, .1, .1, .1, "cm"))) +
  theme(legend.position = "none") +
  scale_x_continuous(labels = scaleFUN1, breaks=c(0,1,2))
```

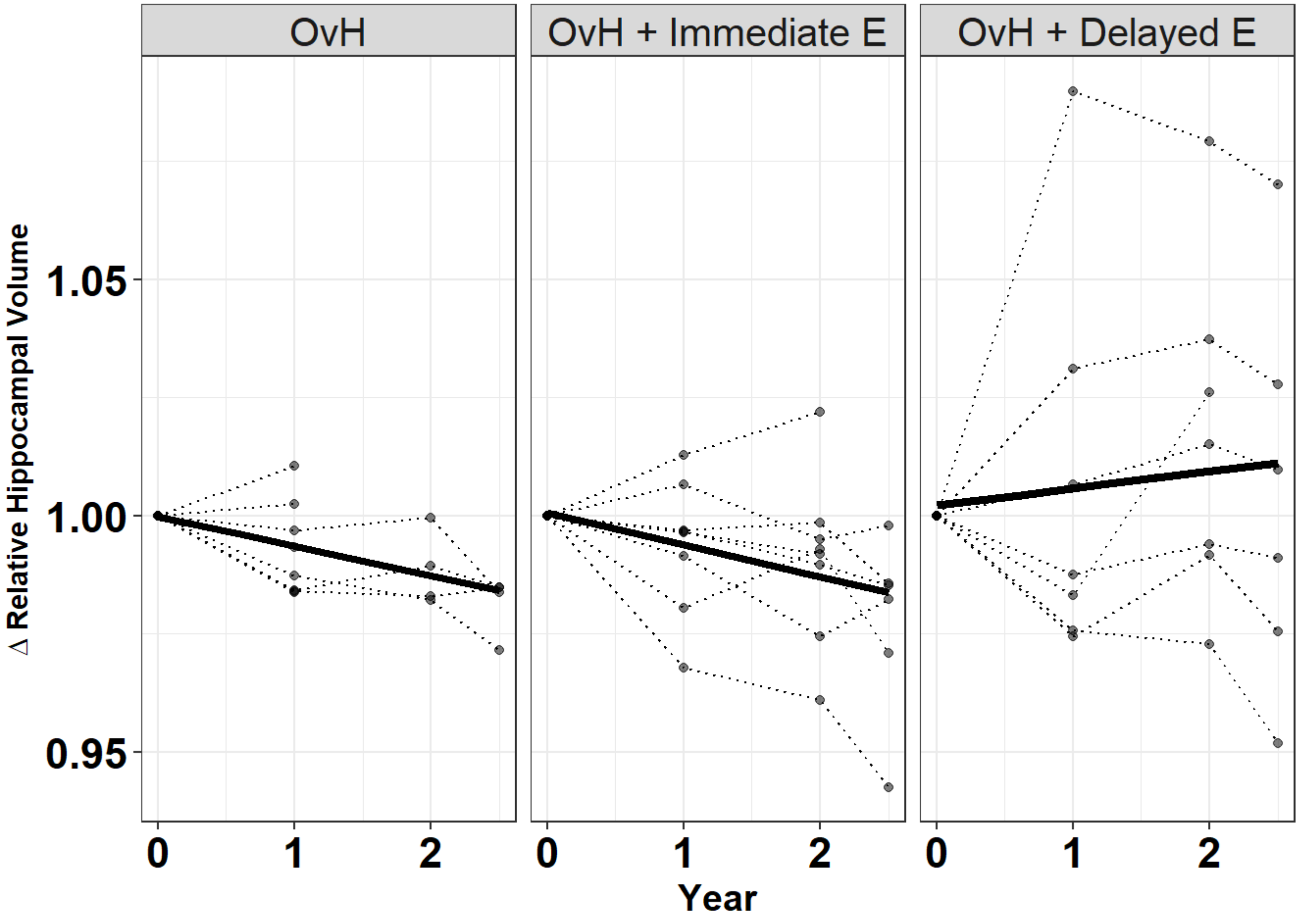

```
#dev.off()
```

It seemed evident from the figure that the Time*Treatment effect for OvH + Delayed E was driven by earlier session data, before the hormone replacment began at year 2. Therefore, we also analyzed an ANOVA for group differences in relative hippocampal volumes using only the final imaging time point.

```
rel_hipp_last_scan_fit <- aov(rel_hipp ~ Group, data = last_scan_data)
summary(rel_hipp_last_scan_fit)
```

```
##             Df    Sum Sq   Mean Sq F value Pr(>F)
## Group        2 2.183e-06 1.092e-06   2.106  0.161
## Residuals   13 6.739e-06 5.184e-07
## 7 observations deleted due to missingness
```
